# Cleft lip and palate: series of unusual clinical cases

**DOI:** 10.1590/S1808-86942010000500019

**Published:** 2015-10-22

**Authors:** Lívia Máris Ribeiro Paranaíba, Roseli Teixeira de Miranda, Daniella Reis Barbosa Martelli, Paulo Rogério Ferreti Bonan, Hudson de Almeida, Julian Miranda Orsi Júnior, Hercílio Martelli Júnior

**Affiliations:** 1 MSc in Stomatology -State University of Campinas - Unicamp; PhD student in Stomatology - Unicamp; 2 PhD in Oral Diagnosis - University of São Paulo - USP, Professor of Stomatology - University of Alfenas; 3 MSc in Health Sciences -State University of Montes Claros - Unimontes, Professor of Semiology - Unimontes; 4 PhD in Stomatology - State University of Campinas - Unicamp, Adjunct Professor - State University of Montes Claros - Unimontes; 5 Resident Physician in Plastic Surgery - Professor at the Medical Sciences School - University of Alfenas; 6 MSc in Health - University of Alfenas; Professor at the Dentistry School - University of Alfenas; 7 PhD; Full Professor. Centro de Ciências Biológicas e da Saúde (CCBS) - Universidade Estadual de Montes Claros, Unimontes, Minas Gerais, Brasil. Campus Universitário Darcy Ribeiro, s/n Vila Mauricéia Montes Claros MG 39400-000. Fundação de Amparo à Pesquisa do Estado de Minas Gerais - Fapemig

**Keywords:** congenital abnormalities, cleft lip, cleft palate.

## Abstract

**Abstract:**

Cleft lip and/or palate (CL/P) represent the most common congenital anomalies of the face, corresponding to approximately 65% of all malformations of the craniofacial region.

**Aim:**

to describe unusual clinical cases of non-syndromic CL/P (CL/PNS), diagnosed in a reference service in Minas Gerais, Brazil, and correlate these alterations with possible risk factors.

**Materials and Methods:**

we carried out a retrospective study, between the years of 1992 and the 1st half of 2009, from medical records.

**Results:**

Among the 778 cases of CL/PNS diagnosed in the period of 17 years, 5 (0.64%) were unusual CL/PNS, and all patients were male. It was found that among the 5 patients, 2 had incomplete right cleft lip with incomplete cleft palate, 2 were affected by left incomplete cleft lip and incomplete cleft palate, and 1 had a cleft lip and palate associated with complete right cleft palate. Risk factors such as consanguinity, maternal smoking and alcohol consumption, medication usage during pregnancy, history of abortion and/or stillbirths and maternal diseases were not associated with unusual CL/PNS.

**Conclusions:**

This study described 5 unusual cases of CL/PNS in a Brazilian population; no associations with the risk factors analyzed were seen. It also confirmed the unusualness of the prevalence of such alterations.

## INTRODUCTION

Cleft lip and/or palate (CL/P) (OMIM 119530) represents the most common of the congenital facial anomalies, making up approximately 65% of all craniofacial malformations^1^. The incidence of CL/P is approximately 1 in every 500-2,000 live births, varying according to geographic location, race and the very social and economic situation of the population studied^2^,^3^. In Brazil, there are only a handful of studies as to the incidence of CL/P, and they vary considerably. According to Brazilian epidemiological surveys, the incidence of CL/P varies between 0.19 to 1.54 for every 1,000 births^4^, ^5^, ^6^. It is not known whether this epidemiological difference is real or associated with methodological differences^6^.

70% of the affected individuals develop non-syndromic CL/P, in other words, without association with other malformations and without behavioral and/or cognitive changes. The remaining 30% are associated with Mendelian disorders (dominant autosomal, recessive autosomal or xlinked), chromosomal, teratogenic or sporadic conditions which include multiple congenital deffects^7^,^8^. Even being a common congenital defect, CL/P etiopathogeny is still uncertain^9^. This is mostly a reflex of the complexity and diversity of the molecular mechanisms involved in embryogenesis, with the participation of multiple genes and the influence of environmental factors^10^,^11^.

It is widely accepted that CL/P has a multifactorial etiology, with genetic and environmental components. Among the environment risk factors for CL/P we stress: maternal diet and vitamin supplements, alcohol ingestion, smoking, the use of anti-seizure medication in the first quarter of gestation and maternal age^9^,^11^. As to the genetic contribution for CL/P, so far, there are numerous genes investigated, but very few clearly associated with CL/P, such as the PVRL1 (Poliovirus receptor related-1)^12^, TGF-b3 (Transforming growth factor beta 3)^13^, MSX1 (Msh homeobox 1)^14^, TBX22 (T-box 22)^15^, FGFs (Fibroblast growth factor)^16^, PTCH (Patched)^17^, and the IRF^6^ (Interferon regulatory factor 6)^7^.

Clinically speaking, CL/P is classified in four groups based on its location in relation to the incisive foramen, as follows: pre-foramen clefts, or simply: labial fissures (LF), post-foramen fissures (PF), trans-foramen fissures or lip-palate fissures (LPF), and rarely facial fissures^18^. The limited knowledge on the very etiology of CL/P makes it difficult even to describe and to distinguish between the varied forms of presentation of these malformations.

Thus, because of the scarcity of national studies, the goal of the present study was to describe and analyze the clinical traits of the uncommon and rare cases of CL/P in a reference center for craniofacial deformities.

## MATERIALS AND METHODS

We carried out a retrospective study between the first semester of 1992 and the 1st semester of 2009, in a reference Ward for craniofacial deformities in the state of Minas Gerais, Brazil. We assessed the clinical charts from the patients seen during this time interval. The fissure distribution is seen on Table 1. We can notice that of the 778 CL/P cases, only 5 (0.64%) were uncommon, which had an anatomical behavior different from that of usual classifications, in other words, (1) CL: including unilateral or bilateral, complete or incomplete pre-foramen; (2) CLP: including unilateral and bilateral trans-foramen clefts, and pre and post foramen clefts; (3) CP: including all complete or incomplete post foramen clefts and (4) Others: here we find the rare facial clefts^18^. From this scientific investigation we excluded syndromic patients with CL/P or those who had other uncommon disorders associated with the clefts.

**Table 1:** Distribution of patients with unusual non-syndromic cleft lip and palate (NSCL/P) *.

	NSCL/P Type	Age	Skin color	Gender	Consanguinity	Past of Miscarriage/stillbirth	Maternal smoking and drinking	Maternal use of folic acid	Use of medication during pregnancy	Medical problem during pregnancy	Family history of NSCL/P
Case 1	FLDI+FPI	1 year e 8 months	L	M	No	no	yes	no	no	no	no
Case 2	FLPEC+FPDC	3 months	F	M	adopted	adopted	adopted	adopted	adopted	adopted	adopted
Case 3	FLEI+FPI	2 months	L	M	No	no	no	no	no	no	yes (paternal uncle)
Case 4	FLEI+FPI	6 months	F	M	No	no	no	no	no	no	no
Case 5	FLDI+FPI	2 years e 2 months	F	M	No	no	no	no	yes	no	no

^*^ FLDI+FPI: Incomplete right-side cleft lip and incomplete cleft palate; FLPEC+FPCD: complete left-side cleft lip and palate and complete right-side cleft palate, FLEI+FPI: Incomplete left-side cleft lip and incomplete cleft palate.

L: Caucasian, F: African-Descendant.

M: Male

Adopted: patient was adopted and there is no information concerning the risk factors analyzed.

From the clinical charts, besides classifying the CL/P, we also collected the following information: age, gender, a past of consanguinity, maternal smoking and alcohol beverage drinking, the use of medication during pregnancy, use of folic acid in the pre-gestational period and in the first quarter of pregnancy, a past of miscarriages and/or stillbirths, maternal diseases and a Family history of CL/P. all the five patients with unusual CL/P were clinically assessed by two professionals trained in the aforementioned institution. This study was carried out in accordance with the principles established by resolution 196/88 from the National Health Council of the Ministry of Health, besides being approved by the Ethics Committee in Research of our University.

## RESULTS

**Figure 1:**
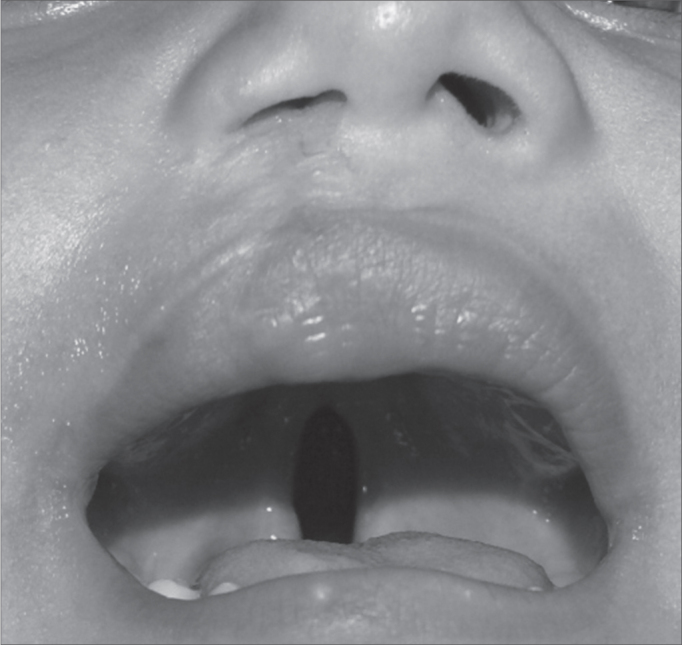
Patient with incomplete right-side cleft lip associated with incomplete cleft palate. Notice that the cleft lip has already been operated.

**Figure 2:**
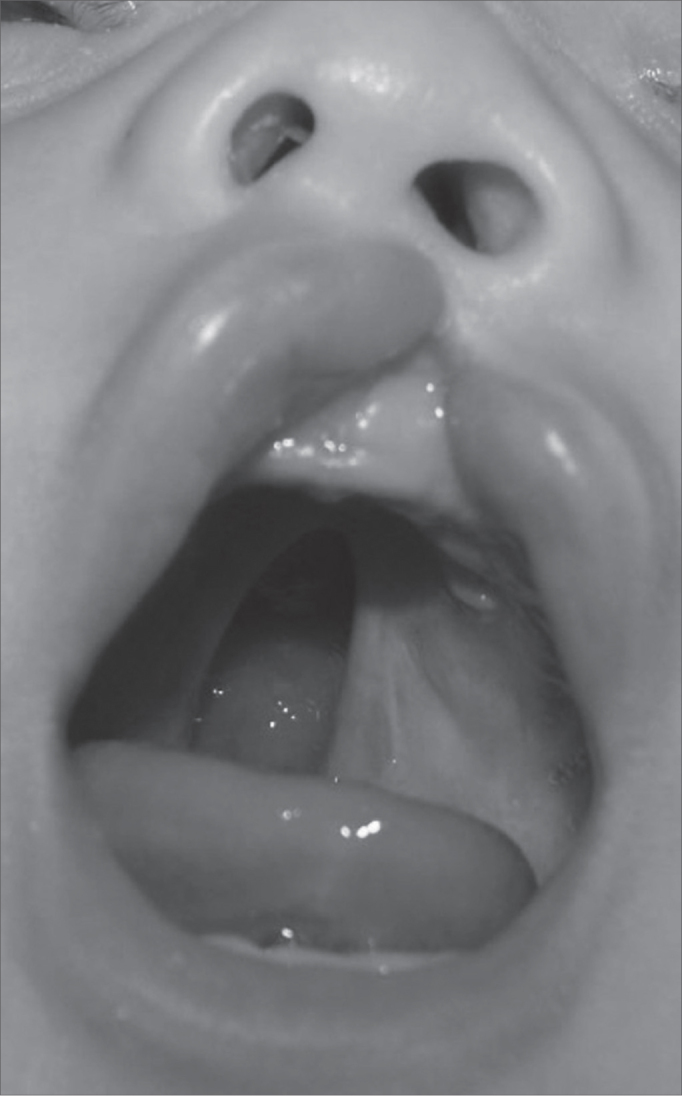
Patient with incomplete left-side cleft lip associated with incomplete cleft palate.

According to Table 1, of the 778 cases of CL/P, seen between the first semester of 1992 and the end of the first semester of 2009, only 5 (0.64%) patients had unusual CL/P. on this same Table we see that all the 5 patients were males and, the mean age at first consultation in visit to our center was 1 year (varying between 2 months and two years and two months). As far as skin color goes, 3 patients were of brown skin and 2 were whites. Considering the type of cleft, 2 patients had incomplete direct cleft lip associated with the incomplete palate cleft (Fig. 1); 2 had incomplete left cleft lip plus incomplete palate cleft (Fig. 2) and 1 had complete left-side palate cleft plus a complete right-side palate cleft.

Mean maternal and paternal ages during gestation in these cases of unusual CL/P was of 26 years for both; we excluded the clinical information from 1 patient who had been adopted, thus making it impossible to collect information. There was no past of consanguinity between the couples, maternal ingestions of alcohol and smoking, use of medication during pregnancy, miscarriage and/or stillbirth past and maternal diseases among the five cases of unusual CL/P we analyzed (Table 1). Nonetheless, one case (case 3) was positive for the family history of CL/P. In such case, a paternal uncle had complete bilateral CL/P. All the five patients, as well as family members, were seen at the health care facility and are currently under multiprofessional clinical follow up.

## DISCUSSION

Numerous epidemiological studies have shown that CL/P has a unique distribution and the incidence of these anomalies varies among the different populations assessed^19^, ^20^, ^21^, ^22^, ^23^. Thus, the Asian population, ancestors of American natives and northern Europeans have a higher incidence of CL/P^1^,^20^ and, contrasting that, Africans and their descendants have a higher incidence of CL alone^20^. In most of the studies, CLP is the most common among NSCL/P^19^,^20^,^23^,^24^ (Non-Syndromic Cleft Lip/Palate) cases. Nonetheless, the prevalence of CL and CP vary according to the population studied^19^,^22^,^25^, ^26^, ^27^. A reduced share of the patients with NSCL/P (1-3.6%) had uncommon forms of bilateral clefts, thus encompassing numerous combinations of CL with different degrees of severity in both sides, such as associations of incomplete CL on one side and complete CLP on the other side^22^,^27^,^28^. Thus, in the present study, because of the scarcity of national studies, uncommon and rare forms of CL/P were described and analyzed.

Of the 778 cases of NSCL/P diagnosed in a Reference Ward in Minas Gerais, Brazil, in the 17 year period, only 5 (0.64%) patients had uncommon forms of these anomalies. A study assessing 803 Brazilian patients who were not operated for CL/P, with or without additional malformations and without recognizable syndromes, found a prevalence of 1.9% of bilateral clefts with unusual associations^22^. In two other studies assessing 835 Mexican patients and 1,669 Iranian patients with CL/P, the uncommon clefts were found in 1% and 3.6% of the cases, respectively^27^,^28^. Nonetheless, differently from these papers analyzed, the present study included only NSCL/P, in other words, without alterations or associated syndromes. Thus, the reduced prevalence found in the present study compared to literature indexes^22^,^27^,^28^ can reflect the methodological difference employed in these populations analyzed.

All the 5 patients affected by unusual NSCL/P were males. Considering the relationship between cleft type and patient gender, most of the studies show that CLP is more frequent in males^29^,^30^. Nonetheless, considering CL and CP alone, the epidemiological investigations showed controversial results^6^,^25^,^27^,^28^. Partially corroborating the results found in the present study, as far as gender is concerned, González et al. (2008)^27^ found the occurrence of unusual CL/P in males, with only one exception.

Bilateral clefts have a relevant morphological variation, with different combinations, but most with limited frequency and, rarely, reported in the literature^22^. The forms of clinical presentation of the unusual NSCL/P found in the present study were, respectively, incomplete left-side CL associated with incomplete CP (2 cases, 40%); direct incomplete CL associated with incomplete CP (2 cases, 40%) and complete left-side CL/P and complete CP (1 case 20%). It is worth noticing that the most severe form of extension of these NSCL/P was associated in only one case.

Comparing the three major types of NSCL/P (CL, CLP and CP), the distribution of unusual CLP by type (unilateral/bilateral), extension (complete/incomplete) and laterality (right/left), is partially in agreement with the literature because of the predominance of incomplete left-side CL, complete left-side CLP and, incomplete CP in the populations investigated^22^,^24^,^25^,^27^.

Among environmental risk-factors for CL/P we stress, consanguinity, smoking, alcohol ingestion, use of medication during pregnancy, insufficient ingestion of folic acid in the pre-gestational period and in the first quarter of pregnancy, a past of miscarriage and/or stillbirth, maternal diseases and family history of clefts^9^,^31^. Nonetheless, in the present scientific investigation we did not find a positive association between these variables and the uncommon NSCL/P. We stress that no mother reported the use of vitamin supplementation or the ingestion of folic acid in the pre-gestational period and/or on the first quarter of pregnancy and only one patient had positive Family history of orofacial clefts. Having in mind the reduced prevalence of unusual NSCL/P in the different populations, the present study confirms the rarity of such malformations in the Brazilian population, besides stressing the importance of describing and assessing these rare cases in an attempt to better understand their etiopathogeny.

## CONCLUSION

The present study assessed 5 rare or unusual cases of NSCL/P in a Brazilian population and confirmed the limited prevalence of such alterations. The clinical types of these fissures were incomplete unilateral lip fissure associated to incomplete palate fissure and complete unilateral cleft lip and palate and complete cleft palate, and they were all seen in male individuals. Moreover, the unusual NSCL/P cases were not associated to the risk factors evaluated. Investigations concerning the rare forms of NSCL/P may enable a better understanding of the etiopathogeny of orofacial clefts.

## ACKNOWLEDGMENT

Fundação de Amparo à Pesquisa do Estado de Minas Gerais (Fapemig) and Conselho Nacional de Desenvolvimento Científico e Tecnológico (CNPq) (HMJ).
